# Assessing the Generalizability of Deep Learning Models Trained on Standardized and Nonstandardized Images and Their Performance Against Teledermatologists: Retrospective Comparative Study

**DOI:** 10.2196/35150

**Published:** 2022-09-12

**Authors:** Ayooluwatomiwa I Oloruntoba, Tine Vestergaard, Toan D Nguyen, Zhen Yu, Maithili Sashindranath, Brigid Betz-Stablein, H Peter Soyer, Zongyuan Ge, Victoria Mar

**Affiliations:** 1 School of Public Health and Preventive Medicine Monash University Melbourne, Victoria Australia; 2 Monash Medical Artificial Intelligence, Monash University, Clayton Melbourne Australia; 3 Department of Dermatology and Allergy Centre Odense University Hospital Odense Denmark; 4 Monash eResearch Centre, Monash University, Clayton, Victoria Melbourne Australia; 5 Central Clinical School, Faculty of Medicine, Nursing and Health Sciences, Monash University Melbourne Australia; 6 Dermatology Research Centre The University of Queensland Diamantina Institute The University of Queensland Brisbane Australia; 7 Airdoc-Monash Research, Monash University, Clayton Melbourne Australia; 8 NVIDIA Artificial Intelligence Tech Centre, Monash University, Clayton, Victoria Melbourne Australia; 9 Victorian Melanoma Service, Alfred Health Melbourne Australia

**Keywords:** artificial intelligence, AI, convolutional neural network, CNN, teledermatology, standardized Image, nonstandardized image, machine learning, skin cancer, cancer

## Abstract

**Background:**

Convolutional neural networks (CNNs) are a type of artificial intelligence that shows promise as a diagnostic aid for skin cancer. However, the majority are trained using retrospective image data sets with varying image capture standardization.

**Objective:**

The aim of our study was to use CNN models with the same architecture—trained on image sets acquired with either the same image capture device and technique (standardized) or with varied devices and capture techniques (nonstandardized)—and test variability in performance when classifying skin cancer images in different populations.

**Methods:**

In all, 3 CNNs with the same architecture were trained. CNN nonstandardized (CNN-NS) was trained on 25,331 images taken from the International Skin Imaging Collaboration (ISIC) using different image capture devices. CNN standardized (CNN-S) was trained on 177,475 MoleMap images taken with the same capture device, and CNN standardized number 2 (CNN-S2) was trained on a subset of 25,331 standardized MoleMap images (matched for number and classes of training images to CNN-NS). These 3 models were then tested on 3 external test sets: 569 Danish images, the publicly available ISIC 2020 data set consisting of 33,126 images, and The University of Queensland (UQ) data set of 422 images. Primary outcome measures were sensitivity, specificity, and area under the receiver operating characteristic curve (AUROC). Teledermatology assessments available for the Danish data set were used to determine model performance compared to teledermatologists.

**Results:**

When tested on the 569 Danish images, CNN-S achieved an AUROC of 0.861 (95% CI 0.830-0.889) and CNN-S2 achieved an AUROC of 0.831 (95% CI 0.798-0.861; standardized models), with both outperforming CNN-NS (nonstandardized model; *P*=.001 and *P*=.009, respectively), which achieved an AUROC of 0.759 (95% CI 0.722-0.794). When tested on 2 additional data sets (ISIC 2020 and UQ), CNN-S (*P*<.001 and *P*<.001, respectively) and CNN-S2 (*P*=.08 and *P*=.35, respectively) still outperformed CNN-NS. When the CNNs were matched to the mean sensitivity and specificity of the teledermatologists on the Danish data set, the models’ resultant sensitivities and specificities were surpassed by the teledermatologists. However, when compared to CNN-S, the differences were not statistically significant (sensitivity: *P*=.10; specificity: *P*=.053). Performance across all CNN models as well as teledermatologists was influenced by image quality.

**Conclusions:**

CNNs trained on standardized images had improved performance and, therefore, greater generalizability in skin cancer classification when applied to unseen data sets. This finding is an important consideration for future algorithm development, regulation, and approval.

## Introduction 

Skin cancer (melanoma and keratinocyte cancer) is the most common type of cancer in fair-skinned populations, with the overall incidence and prevalence increasing worldwide [1]. In an effort to improve current prevention and detection practices, artificial intelligence (AI) has shown promise, at least in experimental settings.

In recent years, advances in machine learning and deep learning have led to increases in the research and exploration of potential applications in dermatology [2-6]. These advancements have led to the production of systems that can diagnose skin conditions through image analysis. With the help of clinical and dermoscopic images for training, convolutional neural networks (CNNs) have been able to compete and even outperform experienced dermatologists when diagnosing and classifying skin cancer [7-11].

Although these models perform well, they are often tested on images that they have already seen or come from the same data set in which the models were trained on, leading to an inflation in their performance [12]. When tested on externally sourced images, the performance of these models is reduced significantly, highlighting the models’ poor generalizability [13].

Generalizability is an important factor that deserves careful consideration when assessing dermatology models. Generalizability refers to how well a model can apply the concepts it has learned from the available training data and implement these same concepts to data it has not seen before.

The method for collecting dermatology image data sets can be defined as nonstandardized and standardized. Nonstandardized image collection refers to images taken using multiple image capture devices and techniques. This method exposes the model to variation in image quality parameters, such as sharpness, brightness, polarization, magnification, color, and distance from lesion (for macroscopic images). Standardized image collection refers to images taken with the same image capture device and technique, resulting in a greater uniformity of images across a data set. It is unknown the extent to which uniformity (or lack thereof) of training images will affect the performance of the resultant CNN model.

Dermatology image data sets are generally not standardized and often collected retrospectively and contain images collected with a variety of techniques and technologies. Theoretically, this variety increases the adaptability of the model and its ability to handle noisy and poorer quality data, thus increasing generalizability. However, with standardized image data sets, there is an expectation for greater consistency in image quality and, therefore, greater performance of the model. When considering the eventual implementation of a CNN model in a clinical setting, it is vital that the model’s performance is impacted minimally by changes to the environment and patient demographic and variation in the presentation of disease. Identifying the factors that affect generalizability will increase the effectiveness of AI model implementation in practice. This retrospective comparative study assessed the generalizability of CNN models trained on standardized and nonstandardized images.

## Methods

### Test Sets, Study Population, and Image Selection

In this study, we compared the performance of CNNs trained on standardized and nonstandardized images when classifying skin cancer as malignant or benign on 3 separate external data sets.

### Ethics Approval

This retrospective comparative study was approved by the Monash University Human Ethics Committee (Project ID 28130).

### Architecture and Training of CNN Models

**Figure 1:**
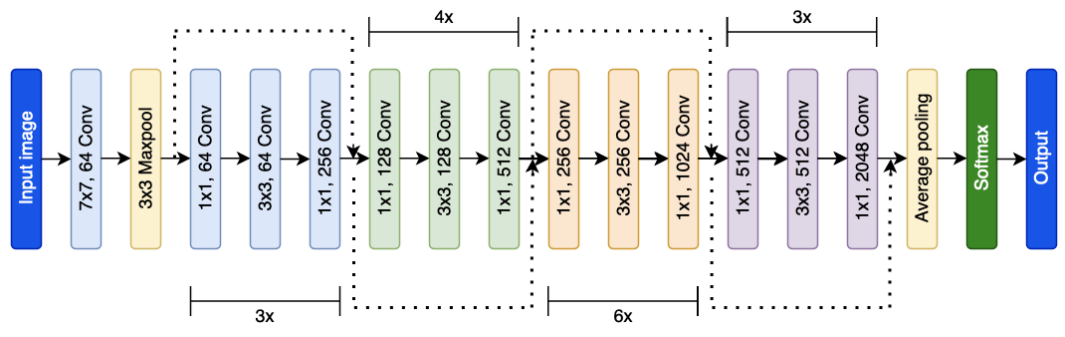
ResNet-50 backbone used by the CNN-NS, CNN-S and CNN-S2 models. CNN: convolutional neural network; Conv: convolutional layers; NS: nonstandardized; S: standardized.

In all, 3 CNN models with the same architecture were trained on International Skin Imaging Collaboration (ISIC) 2019 [14-17] and MoleMap (MoleMap NZ Limited) [2] data sets. Model architecture used ImageNet pretrained ResNet-50 as a backbone (Figure 1) combined with a transformer [18,19]. The ResNet-50 backbone was incorporated because of the trade-off between accuracy and complexity. A transformer was also added to the model to overcome the limitation of CNN in the context of learning global images. The same 3 CNN models were then additionally trained with a ResNet-18 backbone on either the ISIC 2019 (CNN nonstandardized [CNN-NS]) or MoleMap (CNN standardized [CNN-S] and CNN standardized number 2 [CNN-S2]) data sets.

**Figure 2:**
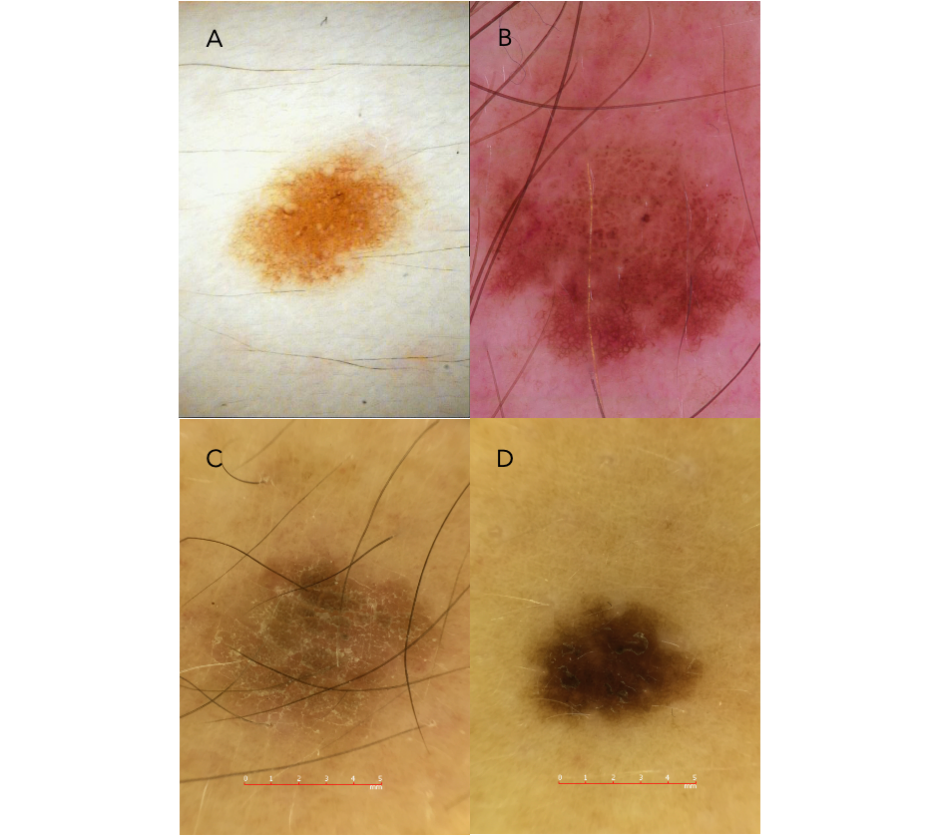
Examples of standardized and nonstandardized images. Images A and B are nonstandardized images, taken using different image capture devices. Images C and D are standardized images, taken using the same image capture device.

CNN-NS was trained on 25,331 nonstandardized ISIC dermoscopic images consisting of 8 skin conditions (Table 1). We define nonstandardized images as images that are taken using multiple image capture technologies (Figure 2). CNN-S was trained on 177,475 standardized, teledermatologist-verified, clinical, and dermoscopic MoleMap images. This data set includes a total of 65 skin conditions organized into a 3-level hierarchical semantic tree (Table 1). This model was trained on standardized images taken using the same camera (DermLite FOTO System). CNN-S2 was trained on 25,331 standardized, teledermatologist-verified, and dermoscopic MoleMap images consisting of 8 skin conditions (Table 1). CNN-NS and CNN-S2 were trained on the same number of images and skin conditions, only differing in the standardization of the images the models were trained on.

**Table 1:** Number of relevant skin diseases the CNN^a^ models were trained on.

Skin disease	CNN-NS^b^, n	CNN-S^c^, n	CNN-S2^d^, n
Melanoma	4522	11,796	4522
Benign naevus	12,875	66,891	12,875
Benign keratosis	2624	22,100	2624
Dermatofibroma	239	4440	239
Basal cell carcinoma	3323	22,292	3323
Actinic keratosis and intraepithelial carcinoma	867	40,440	867
Squamous cell carcinoma	628	7060	628
Vascular proliferations	253	2456	253
Total	25,331	177,475	25,331

^a^CNN: convolutional neural network.

^b^CNN-NS: CNN nonstandardized.

^c^CNN-S: CNN standardized.

^d^CNN-S2: CNN standardized number 2.

### Assessment of CNN Performance

CNN performance was assessed using 3 separate test data sets that were not used in model training.

#### Test Set 1

The Danish data set was provided by the Department of Dermatology and Allergy Centre, Odense University Hospital and collected between January 9 and October 31, 2018 [20]. General practitioners from 50 practices across southern Denmark were trained for 1 hour with the image capture equipment required to take images of lesions that are suspicious for malignant melanoma and nonmelanoma skin cancer. A total of 600 images were collected from 519 Danish patients, predominantly involving patients with Fitzpatrick skin types II and III, were used. The “ground truth” diagnosis was achieved by histopathology, follow-up, or a single face-to-face evaluation (308 of the 600 lesions in the original data set were only seen once face-to-face). Images containing clinical features that could not be identified were removed from the data set, leaving 569 images. Lesion classification can be seen in Table 2.

The 569 images were taken using an iPhone 6 smartphone (Apple Inc) and a handyscope (FotoFinder Systems GmbH) with an overview, a close-up, and a dermoscopic image being taken of the lesions.

In total, 4 dermatologists were involved in the face-to-face and teledermatology evaluations of the 519 patients. The quality of the images was rated as “poor,” “fair,” or “good” by 3 allocators. Images were assigned to the different categories when there was agreement between 2 or more allocators. 

**Table 2:** Skin disease breakdown of test sets 1, 2, and 3.

Classification, skin disease			Test set 1 (Danish data set), n		Test set 2 (UQ^a^ data set), n		Test set 3 (ISIC^b^ 2020 data set), n
**Malignant**							
	Melanoma	20		21		584	
	Basal cell carcinoma	80		72		N/A^c^	
	Squamous cell carcinoma	5		7		N/A	
	Actinic keratosis and intraepithelial carcinoma	50		65		N/A	
	Other malignancy	3		N/A		N/A	
**Benign**							
	Benign keratosis	115		64		179	
	Vascular proliferations	45		1		N/A	
	Other	95		22		27,170	
	Benign naevus	156		170		5193	
Total			569		422		33,126

^a^UQ: The University of Queensland.

^b^ISIC: International Skin Imaging Collaboration.

^c^N/A: not applicable.

#### Test Set 2

The University of Queensland (UQ) data set contained 422 dermoscopic images provided by The University of Queensland, Diamantina Institute, Dermatology Research Centre and captured using the EOS Rebel T6i camera (Canon) and ATBM master automated mole-mapping system (FotoFinder Systems GmbH) between 2016 and 2020, with all lesions diagnosed through histopathology (Table 2)*.*

#### Test Set 3

The ISIC 2020 data set contained 33,126 dermoscopic images provided by the ISIC and collected from 3 continents between 1998 and 2020 [21]*.* The 33,126 images in the ISIC 2020 test set contained 59 images that overlap with the 25,331 images in the ISIC 2019 data set used for the training of CNN-NS.

All 3 test sets were imbalanced, with the Danish data set containing 411 benign and 158 malignant images, the UQ data set containing 257 benign and 165 malignant images, and the ISIC 2020 data set containing 27,131 benign and 5995 malignant images, which is reflective of the breakdown seen in a clinical setting. As the classification is binary, the imbalance had no effect on the study. Lesion classification can be seen in Table 2.

### Statistical Analysis

Statistical analysis was performed using Python software (version 3.8.13; Python Software Foundation) and Stata statistical software (version SE 17; StataCorp). The primary outcome measures were sensitivity, specificity, and area under the receiver operating characteristic curve (AUROC) for the binary classification of lesions.

For each input image, the CNNs provided a score between 0 and 1 representing the probability that the input image is malignant. In binary classifications, thresholds are applied to the CNN models to establish the point at which an input image is labeled malignant. This threshold is variable and allows for the manipulation of the sensitivity and specificity of the models.

The performance was assessed by aligning the sensitivity and specificity of the CNN models to the teledermatologists’ and by calculating the AUROC. AUROC allows for the direct comparison of different models regardless of the threshold applied. Delong nonparametric test was used to evaluate the statistical difference between AUROC values resulting from the same data set. Additionally, 95% CI for the AUROC was computed using 2000 stratified bootstrap replicates. McNemar test was used to compare the sensitivities and specificities of the CNN models. The 1-sample, 2-tailed *t* test was used to compare the mean sensitivities and specificities of the teledermatologists against the sensitivities and specificities of the CNN models. *P* values <.05 were considered to have statistically significant differences.

## Results

### Model Validation

**Figure 3:**
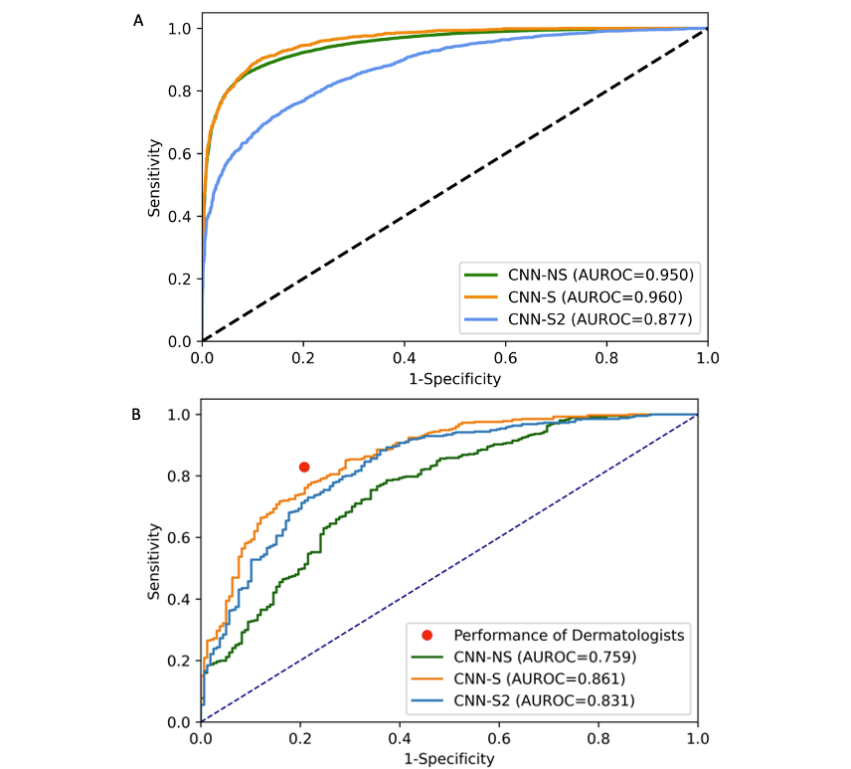
Receiver operating characteristic curves and AUROC for (A) the 3 CNN models during training and (B) the performances of the teledermatologists and the 3 CNN models on the Danish test set. The receiver operating characteristic curves and AUROC of the CNN models in relation to the sensitivity and 1-specificity of the teledermatologists were tested on the 569 Danish test images. The teledermatologists' performance was greater than all of the CNN models. AUROC: area under the receiver operating characteristic curve; CNN: convolutional neural network; NS: nonstandardized; S: standardized.

During training, each model was internally validated on their training images. The model trained on nonstandardized images (CNN-NS) showed an AUROC of 0.950, whereas both models trained on standardized images (CNN-S and CNN-S2) showed an AUROC of 0.960 and 0.877, respectively (Figure 3).

### CNN Performance on Test Set 1

Each CNN model was tested on the externally sourced Danish test set of 569 images. CNN-NS performance fell with an AUROC of 0.759 (95% CI 0.714-0.802). CNN-S outperformed CNN-NS when examined on the Danish test set, with an AUROC of 0.861 (95% CI 0.828-0.894), showing significantly greater generalizability than CNN-NS (*P*=.001; Figure 3). CNN-S2, the standardized model trained on the same number of images as CNN-NS, also outperformed the model, showing an AUROC of 0.831 (95% CI 0.789-0.869; *P*=.009). Among the standardized models, CNN-S had the greatest AUROC (0.861 vs 0.831; *P*=.06).

### CNN Performance on Test Set 2

**Figure 4:**
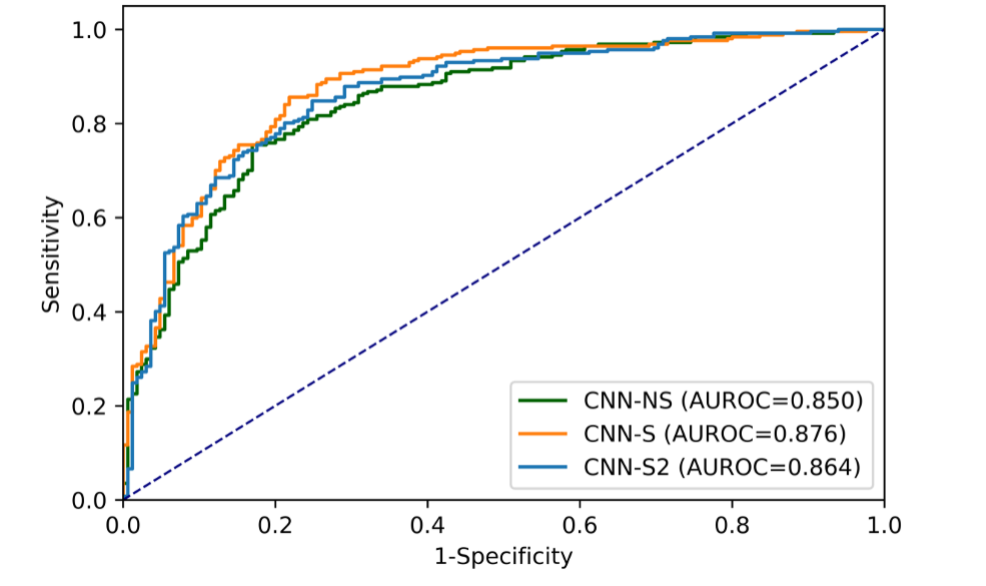
Receiver operating characteristic curves and AUROC for the 3 CNN models on The University of Queensland test set. AUROC: area under the receiver operating characteristic curve; CNN: convolutional neural network; NS: nonstandardized; S: standardized.

When tested on the externally sourced UQ test set of 422 images, CNN-NS performed well with an AUROC of 0.850 (95% CI 0.812-0.887). CNN-S outperformed CNN-NS when tested on the UQ image set, with an AUROC of 0.876 (95% CI 0.842-0.911), again showing greater generalizability than CNN-NS (*P*=.08; Figure 4). CNN-S2 also achieved a slightly greater AUROC (0.864, 95% CI 0.828-0.900) compared to CNN-NS, though this was not statistically significant (*P*=.35). Among the standardized models, CNN-S had the greatest AUROC (0.8765 vs 0.8638), though the difference was not statistically significant (*P*=.23).

### CNN Performance on Test Set 3

**Figure 5:**
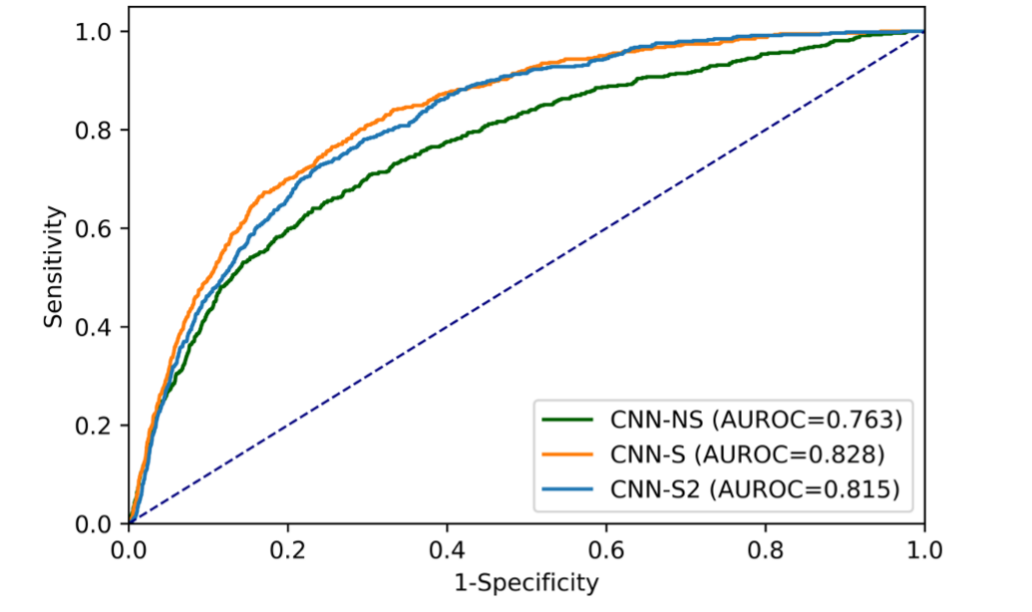
Receiver operating characteristic curves and AUROC for the 3 CNN models on the International Skin Imaging Collaboration 2020 test set. AUROC: area under the receiver operating characteristic curve; CNN: convolutional neural network; NS: nonstandardized; S: standardized.

When tested on the publicly available ISIC 2020 test set of 33,126 images, the performance of CNN-NS was reduced, with an AUROC of 0.763 (95% CI 0.743-0.783). CNN-S significantly outperformed CNN-NS when examined on the ISIC test set (*P*<.001), with an AUROC of 0.828 (95% CI 0.812-0.843), showing greater generalizability than CNN-NS (Figure 5). CNN-S2 also significantly outperformed the CNN-NS (*P*<.001), with an AUROC of 0.815 (95% CI 0.799-0.830).

### Teledermatologist Versus CNN Performance in Test Set 1

Teledermatologists (N=4) were split into 2 groups, teledermatologists 1 and teledermatologists 2. To evaluate the performance of the teledermatologists against the CNN models, we used the mean sensitivity and specificity of the 2 teledermatologist groups as a standard. On the Danish images, the teledermatologists achieved a mean sensitivity of 82.9% (95% CI 80.8%-85.0%) and specificity of 79.2% (95% CI 78.5%-79.9%).

The CNN models’ malignancy threshold score can be manipulated, which can change the sensitivity and specificity of the models. To compare the performance of the models to each other, we first matched the sensitivity to that of the teledermatologists (82.9%). CNN-S achieved a specificity of 72% (95% CI 66.9%-75.9%), outperforming both CNN-S2 (62%, 95% CI 55.7%-65.3%; *P*=.02) and CNN-NS (45%, 95% CI 38.4-49.6; *P*=.001). Additionally, CNN-S2 revealed a greater specificity than CNN-NS (*P*=.001). Next, we matched the specificity of each model to that of the teledermatologists (79.2%). CNN-S showed a sensitivity of 74.7% (95% CI 67.8%-81.8%), outperforming both CNN-S2 (71.5%; 95% CI 63.8%-78.4%; *P*=.77) and CNN-NS (56.3%; 95% CI 48.2%-64.2%; *P*=.006). Additionally, CNN-S2 revealed a greater sensitivity than CNN-NS (*P*=.003).

To compare models’ performance to that of the teledermatologists, we compared the mean sensitivity (82.9%) and specificity (79.2%) of the teledermatologists to that of each model. This comparison revealed that our highest performing model (CNN-S) had a sensitivity (74.7% vs 82.9%; *P*=.10) and specificity (72.0% vs 79.2%; *P*=.053) comparable to that of the teledermatologists (Table 3). However, both CNN-S2 and CNN-NS had significantly lower specificity and CNN-NS had significantly lower sensitivity when compared to the teledermatologists (Table 3).

**Table 3:** Sensitivity and specificity of the CNN^a^ models when matched to the average performance of the teledermatologists.

	Specificity when matched to sensitivity, % (95% CI)	*P* value	Sensitivity when matched to specificity, % (95% CI)	*P* value
Teledermatologists (average)	79.2 (74.82-82.91)	Reference	82.9 (76.1-88.4)	Reference
CNN-S^b^	72 (67.4-76.3)	.053	74.7 (67.2-81.3)	.10
CNN-S2^c^	65.2 (60.4-69.8)	.03	71.5 (63.8-78.4)	.07
CNN-NS^d^	46.7 (41.8-51.7)	.01	56.3 (48.2-64.2)	.03

^a^CNN: convolutional neural network.

^b^CNN-S: CNN standardized.

^c^CNN-S2: CNN standardized number 2.

^d^CNN-NS: CNN nonstandardized.

### Effect of Image Quality on the Performance of Teledermatologists

**Figure 6:**
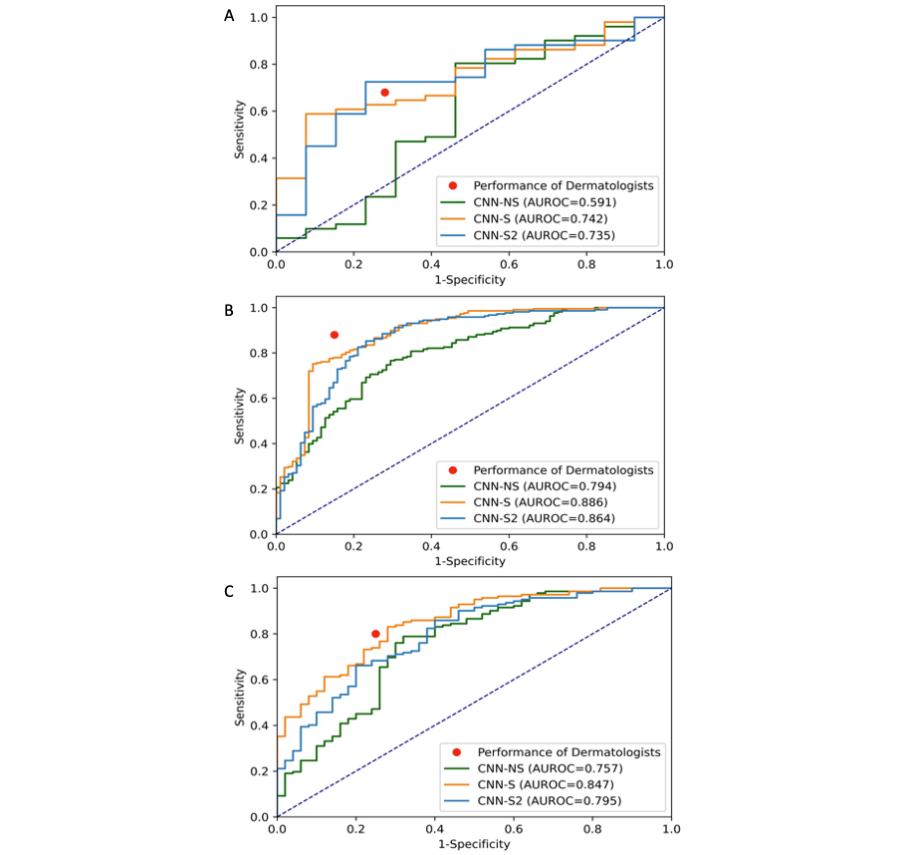
Impact of image quality on the performance of the teledermatologists and AUROC of the CNN Models. The receiver operating characteristic curves and the AUROC of the CNN models and average sensitivity and 1-specificity of the teledermatologists on the Danish test set were split into (A) poor, (B) fair, and (C) good quality images. AUROC: area under the receiver operating characteristic curve; CNN: convolutional neural network; NS: nonstandardized; S: standardized.

When taking the image quality of test set 1 into consideration, the AUROCs of CNN-NS, CNN-S, and CNN-S2 increased as the quality of images improved (Figure 6). CNN-NS showed an AUROC of 0.591 (95% CI 0.389-0.778), 0.757 (95% CI 0.670-0.835), and 0.794 (95% CI 0.741-0.844) for images of poor, fair, and good quality, respectively. CNN-S showed AUROCs of 0.742 (95% CI 0.602-0.864; poor quality), 0.847 (95% CI 0.792-0.879; fair quality), and 0.886 (95% CI 0.817-0.909; good quality), and CNN-S2 showed AUROCs of 0.735 (95% CI 0.578-0.873; poor quality), 0.795 (95% CI 0.721-0.861; fair quality), and 0.864 (95% CI 0.820-0.909; good quality).

## Discussion

### Principal Findings

Our results provide evidence that models trained on standardized images outperform and, hence, achieve greater generalizability than models trained on nonstandardized images. In recent years, advances in machine learning have led to the development of models that can compete and even outperform dermatologists in the classification of skin cancer [7-11]. Although these models have been shown to perform well when tested on a subset of images from their training data set, the generalizability of these models to images taken in different clinical settings and with different devices is unknown.

The impact that the varying image acquisition devices and techniques have on CNN model performance in dermatology has not been explored in the literature to date; however, the lack of imaging standardization in dermatology has been highlighted. The collection, transfer, and storage of clinical and dermoscopic images are not standardized in dermatology and have implications on the creation of data sets for machine learning, the reproducibility of imaging, and accessibility to relevant metadata for the images [22,23].

The standardized models (CNN-S and CNN-S2) consistently outperformed the nonstandardized model (CNN-NS) on all test sets. The statistical significance was directly affected by the number of images in the 3 test sets, with fewer images in test set 2 resulting in a nonsignificant difference in performance. Larger test sets will have a more accurate measure of model performance, and this finding would need to be considered when reporting validation results.

The ISIC holds an annual challenge that invites contestants to create a model that is trained and tested on images provided by the ISIC. In the AI community, the model that wins the ISIC challenge often holds a reputation as one of the best available. However, if tested on external data, the same performance is not guaranteed. If models are both trained and tested on the same set of images, then they are subjected to overfitting and thus poorer generalizability. The quality of a model should therefore be judged on its performance on multiple external data sets from varying population groups.

Several studies have looked at the performance of CNN models compared to the performance of dermatologists. These models perform comparably and even outperform dermatologists when classifying skin cancers. However, it is important to note that the images used in test sets are often taken from the same data sets used in the training of the models [7-11]. It is important when comparing models to dermatologists that the CNN is externally validated. This validation provides a clearer indication of the performance of the models in comparison to dermatologists and their ability to generalize to external data sets.

In our study, when tested on test set 1, the teledermatologists outperformed all models. Interestingly, CNN-S was trained on Australian and New Zealand patients and generalized well to the Danish images. There was no statistical difference between the sensitivity and specificity of the teledermatologists and the matched sensitivity and specificity of CNN-S. It is important to note that the Danish teledermatologists were predominantly trained on Danish skin and had access to metadata and multiple image viewpoints for a single lesion, which the models did not have access to. Previous studies have shown that the addition of metadata and inclusion of both macroscopic and dermoscopic images of a lesion can improve the performance of the model [24,25]. Therefore, incorporating these features into future models will be important and may level the playing field when assessing performance against teledermatologists’ clinical assessment.

The Danish images used in our study were taken by general practitioners who were required to undertake training to use the image capture technology. However, there were some issues with the quality of the images: some lesions were not centered, several lesions may be present within a single image, and parts of lesions were not included within the image frame. As the image quality improved, the diagnostic performance of all models and teledermatologists also increased. This finding highlights the influence that image capture techniques and image quality can have on CNN models and teledermatologists’ diagnostic ability. This finding is also a consideration when designing models for integration into web-based tools or mobile apps with consumers as end users, as the quality of images taken by consumers on their smartphones will vary, especially in the absence of training.

### Limitations

Our study has several limitations. First, the MoleMap data set used to train our 2 standardized CNN models was labeled by dermatologists; however, only very few images were biopsy proven. Given that histopathology is the gold standard for diagnosis, some of these images may have been mislabeled, which could have an impact on the performance of the models. Second, in test set 1 with 569 images, we only had access to 221 biopsy-proven images. The remaining 348 images in the test set 1 were labeled by dermatologists, which allows for the possibility of mislabeling. Third, the quality of the images in the training data sets (ISIC and MoleMap) and the type of image modality may have played a part in the performance of the models rather than the standardization of the images. It is important to consider that the quality of the camera used in the standardized MoleMap data set is less variable than the nonstandardized ISIC 2019 data set, which may have led to a discrepancy in the performance. CNN-S was trained on a combination of dermoscopic and macroscopic images, whereas CNN-NS and CNN-S2 were trained only on dermoscopic images. This combination of image modalities may have had an influence on the strength of the CNN-S model. Additionally, the models are complex, making it difficult to understand the process behind their decision-making (ie, a black box). This is an important limitation of the models and of this study and will be addressed through the incorporation of explainable AI techniques in our future models. Finally, in test set 1, the number of lesions in each group becomes small when divided into images of poor, fair, and good quality. In future studies, it would be better to evaluate a larger data set split among the quality groups to more confidently assess the relationship between image quality and CNN performance.

### Conclusion 

In this study, CNN models trained on standardized images based on dermoscopic and macroscopic modalities performed better than a CNN model with the same architecture trained on nonstandardized images when tested on external image data sets. This finding has important implications for model generalizability in the binary classification of skin cancer. In test set 1, image quality also had a direct impact on the performance of the models. For future algorithm training, development, and registration, it is important that model generalizability is considered through the evaluation of model performance on external image data sets.

## Abbreviations

AI: artificial intelligence
AUROC: area under the receiver operating characteristic curve
CNN: convolutional neural network
CNN-NS: CNN nonstandardized
CNN-S: CNN standardized
CNN-S2: CNN standardized number 2
ISIC: International Skin Imaging Collaboration
NHMRC: National Health and Medical Research Council
UQ: The University of Queesland

## References

[1] Apalla Z, Lallas A, Sotiriou E, Lazaridou E, Ioannides D (2017). Epidemiological trends in skin cancer. Dermatol Pract Concept.

[2] Ge Z, Demyanov S, Chakravorty R, Bowling A, Garnavi R (2017). Skin disease recognition using deep saliency features and multimodal learning of dermoscopy and clinical images. Lecture Notes in Computer Science, vol 10435.

[3] Gu Y, Ge Z, Bonnington CP, Zhou J (2020). Progressive transfer learning and adversarial domain adaptation for cross-domain skin disease classification. IEEE J Biomed Health Inform.

[4] Lei B, Xia Z, Jiang F, Jiang X, Ge Z, Xu Y, Qin J, Chen S, Wang T, Wang S (2020). Skin lesion segmentation via generative adversarial networks with dual discriminators. Med Image Anal.

[5] Wada M, Ge Z, Gilmore SJ, Mar VJ (2020). Use of artificial intelligence in skin cancer diagnosis and management. Med J Aust.

[6] Felmingham CM, Adler NR, Ge Z, Morton RL, Janda M, Mar VJ (2021). The importance of incorporating human factors in the design and implementation of artificial intelligence for skin cancer diagnosis in the real world. Am J Clin Dermatol.

[7] Esteva A, Kuprel B, Novoa RA, Ko J, Swetter SM, Blau HM, Thrun S (2017). Dermatologist-level classification of skin cancer with deep neural networks. Nature.

[8] Fujisawa Y, Otomo Y, Ogata Y, Nakamura Y, Fujita R, Ishitsuka Y, Watanabe R, Okiyama N, Ohara K, Fujimoto M (2019). Deep-learning-based, computer-aided classifier developed with a small dataset of clinical images surpasses board-certified dermatologists in skin tumour diagnosis. Br J Dermatol.

[9] Tschandl P, Rosendahl C, Akay BN, Argenziano G, Blum A, Braun RP, Cabo H, Gourhant J, Kreusch J, Lallas A, Lapins J, Marghoob A, Menzies S, Neuber NM, Paoli J, Rabinovitz HS, Rinner C, Scope A, Soyer HP, Sinz C, Thomas L, Zalaudek I, Kittler H (2019). Expert-level diagnosis of nonpigmented skin cancer by combined convolutional neural networks. JAMA Dermatol.

[10] Haenssle HA, Fink C, Schneiderbauer R, Toberer F, Buhl T, Blum A, Kalloo A, Hassen ABH, Thomas L, Enk A, Uhlmann L, Alt Christina, Arenbergerova Monika, Bakos Renato, Baltzer Anne, Bertlich Ines, Blum Andreas, Bokor-Billmann Therezia, Bowling Jonathan, Braghiroli Naira, Braun Ralph, Buder-Bakhaya Kristina, Buhl Timo, Cabo Horacio, Cabrijan Leo, Cevic Naciye, Classen Anna, Deltgen David, Fink Christine, Georgieva Ivelina, Hakim-Meibodi Lara-Elena, Hanner Susanne, Hartmann Franziska, Hartmann Julia, Haus Georg, Hoxha Elti, Karls Raimonds, Koga Hiroshi, Kreusch Jürgen, Lallas Aimilios, Majenka Pawel, Marghoob Ash, Massone Cesare, Mekokishvili Lali, Mestel Dominik, Meyer Volker, Neuberger Anna, Nielsen Kari, Oliviero Margaret, Pampena Riccardo, Paoli John, Pawlik Erika, Rao Barbar, Rendon Adriana, Russo Teresa, Sadek Ahmed, Samhaber Kinga, Schneiderbauer Roland, Schweizer Anissa, Toberer Ferdinand, Trennheuser Lukas, Vlahova Lyobomira, Wald Alexander, Winkler Julia, Wölbing Priscila, Zalaudek Iris, Reader study level-I and level-II Groups (2018). Man against machine: diagnostic performance of a deep learning convolutional neural network for dermoscopic melanoma recognition in comparison to 58 dermatologists. Ann Oncol.

[11] Han SS, Kim MS, Lim W, Park GH, Park I, Chang SE (2018). Classification of the clinical images for benign and malignant cutaneous tumors using a deep learning algorithm. J Invest Dermatol.

[12] Dick V, Sinz C, Mittlböck Martina, Kittler H, Tschandl P (2019). Accuracy of computer-aided diagnosis of melanoma: a meta-analysis. JAMA Dermatol.

[13] Navarrete-Dechent C, Dusza S, Liopyris K, Marghoob A, Halpern A, Marchetti M (2018). Automated dermatological diagnosis: hype or reality?. J Invest Dermatol.

[14] Tschandl Philipp, Rosendahl Cliff, Kittler Harald (2018). The HAM10000 dataset, a large collection of multi-source dermatoscopic images of common pigmented skin lesions. Sci Data.

[15] Codella NCF, Gutman D, Celebi ME, Helba B, Marchetti MA, Dusza SW, Kalloo A, Liopyris K, Mishra N, Kittler H, Halpern A (2017). Skin lesion analysis toward melanoma detection: a challenge at the 2017 International Symposium on Biomedical Imaging (ISBI), hosted by the International Skin Imaging Collaboration (ISIC). arXiv.

[16] Combalia M, Codella NCF, Rotemberg V, Helba B, Vilaplana V, Reiter O, Carrera C, Barreiro A, Puig S, Malvehy J (2019). BCN20000: dermoscopic lesions in the wild. arXiv.

[17] ISIC Challenge datasets. ISIC Challenge.

[18] Carion N, Massa F, Synnaeve G, Usunier N, Kirillov A, Zagoruyko S (2020). End-to-end object detection with transformers. Lecture Notes in Computer Science, vol 12346.

[19] He K, Zhang X, Ren S, Sun J (2016). Deep residual learning for image recognition.

[20] Vestergaard T, Prasad S, Schuster A, Laurinaviciene R, Andersen M, Bygum A (2020). Diagnostic accuracy and interobserver concordance: teledermoscopy of 600 suspicious skin lesions in Southern Denmark. J Eur Acad Dermatol Venereol.

[21] Rotemberg V, Kurtansky N, Betz-Stablein B, Caffery L, Chousakos E, Codella N, Combalia M, Dusza S, Guitera P, Gutman D, Halpern A, Helba B, Kittler H, Kose K, Langer S, Lioprys K, Malvehy J, Musthaq S, Nanda J, Reiter O, Shih G, Stratigos A, Tschandl P, Weber J, Soyer HP (2021). A patient-centric dataset of images and metadata for identifying melanomas using clinical context. Sci Data.

[22] Eapen BR, Kaliyadan F, Ashique KT (2022). DICODerma: a practical approach for metadata management of images in dermatology. J Digit Imaging.

[23] Caffery LJ, Rotemberg V, Weber J, Soyer HP, Malvehy J, Clunie D (2020). The role of DICOM in artificial intelligence for skin disease. Front Med (Lausanne).

[24] Höhn Julia, Hekler A, Krieghoff-Henning E, Kather JN, Utikal JS, Meier F, Gellrich FF, Hauschild A, French L, Schlager JG, Ghoreschi K, Wilhelm T, Kutzner H, Heppt M, Haferkamp S, Sondermann W, Schadendorf D, Schilling B, Maron RC, Schmitt M, Jutzi T, Fröhling Stefan, Lipka DB, Brinker TJ (2021). Integrating patient data into skin cancer classification using convolutional neural networks: systematic review. J Med Internet Res.

[25] Yap J, Yolland W, Tschandl P (2018). Multimodal skin lesion classification using deep learning. Exp Dermatol.

